# The Complexity of Familial Inheritance in Pectus Excavatum: A Ten-Family Exome Sequencing Analysis

**DOI:** 10.3390/genes15111429

**Published:** 2024-11-01

**Authors:** Juan M. Farina, Rory J. Olson, Radhika Dhamija, Anne Bofferding, Aleksandar Sekulic, Jan B. Egan, Dawn E. Jaroszewski

**Affiliations:** 1 Department of Cardiovascular and Thoracic Surgery, Mayo Clinic, Phoenix, AZ 85054, USA; 2 Center for Individualized Medicine, Mayo Clinic, Rochester, MN 55905, USA; 3 Department of Clinical Genomics and Neurology, Mayo Clinic, Rochester, MN 55905, USA; dhamija.radhika@mayo.edu; 4 Center for Individualized Medicine, Mayo Clinic, Phoenix, AZ 85054, USA; 5 City of Hope Cancer Center, Goodyear, AZ 85338, USA; 6 Translational Genomics Research Institute, Phoenix, AZ 85004, USA

**Keywords:** pectus excavatum, exome sequencing, genetics

## Abstract

**Background/Objectives**: Pectus excavatum (PEx) is considered, at least partially, a familial disorder. A variety of inheritance patterns, associations with genetic syndromes, and pathogenic variants have been reported. However, the etiology of this condition is still not completely understood, and no known genes have been identified as definitive contributors. **Methods**: Family members with a confirmed PEx diagnosis (one proband and two first-degree relatives) and non-affected members were recruited into this study. Exome sequencing was performed on all affected familial PEx cases to systematically screen for candidate genes that are likely to be causative for PEx, and on non-affected family members for variant segregation analysis. **Results**: Ten families, with three affected members each, participated, providing thirty familial PEx cases. Different inheritance patterns were represented across the ten pedigrees, with possible incomplete penetrance. Genetic variants in *REST* (essential for neuronal development and associated with pectus deformities in prior studies), *SMAD4* (variants can predispose individuals to thoracic aortic diseases), and *COL5A* (associated with Ehlers–Danlos syndrome and Fibromuscular dysplasia) were initially identified as potentially linked to the development of pectus deformities and segregated with the phenotype. No variants were shared across families in the studied population. **Conclusions**: Germline exome sequencing of families with multiple individuals affected by PEx in our study identified potential gene candidates linked to PEx. These candidates are private to individual families and no strong candidates shared across multiple families were identified. These findings suggest that the inheritance of PEx may not be strongly related to a shared single genetic variant in known genes. Given the accumulating evidence for the genetic basis of familial PEx, further studies, including polygenic analyses, as well as assessment of the non-coding genome and possible epigenetic markers are warranted.

## 1. Introduction

Pectus excavatum (PEx) is the most common congenital chest wall deformity, with an estimated prevalence of 1 in 300–1000 live births [1,2]. In this condition, the sternum is displaced posteriorly to produce a funnel-shaped depression. There have been several proposed theories for the development of PEx, with the most common including abnormalities of the diaphragm muscle resulting in posterior traction on the sternum and the intrinsic failure of osteogenesis and/or chondrogenesis of the anterior chest wall [3].

The etiology of PEx has remained elusive, although clinical experience and the prior literature support the conclusion that PEx is, at least partially, a familial disorder often seen in more than one sibling and in multiple generations. It is estimated that ~40% of PEx patients have affected family members with similar congenital deformities [3,4]. Prior familial studies have shown a variety of inheritance patterns, including autosomal dominant, autosomal recessive, and X-linked [3]. Associations of PEx with genetic disorders such as Marfan syndrome, Loeys–Dietz syndrome, and Ehlers–Danlos, among others, have also been reported. Despite prior efforts, no definite genetic contributor to PEx development has been identified [5]. However, prior larger studies in this field mainly relied on low-resolution approaches, and those utilizing exam sequencing were focused on reporting isolated family cases of PEx [3,5,6]. To date, no study has reported a comprehensive genomic analysis of the exome across a cohort of numerous PEx families.

Identification of the genetic variants that cause PEx may have implications regarding comorbidities and reproductive choices. This could allow earlier diagnosis, with prompt care for patients before PEx impedes cardiopulmonary function. This study was performed to identify genetic variants in PEx patients by performing a genetic analysis in the largest-to-date cohort of families with multiple members affected by this condition.

## 2. Materials and Methods

Families consisting of three members with a confirmed PEx diagnosis (one proband and two first-degree relatives) were identified in the electronic database of a referral center for the treatment of PEx (Mayo Clinic, Phoenix, AZ, USA) and included in this study. PEx cases and unaffected cases were confirmed by using clinical and imaging diagnosis. The severity of PEx was determined using the Computed Tomography-derived Haller Index, which was calculated by measuring the transverse diameter of the chest divided by the sagittal measurement from the sternum to the vertebral body [7]. PEx diagnosis was considered when the Haller Index ≥ 3.25 [7]. Informed consent was obtained from all the participants and approval was obtained from the Mayo Clinic IRB to conduct this research.

As a first phase of the analysis, exome sequencing (ES) was performed on all affected familial PEx cases to systematically screen for the candidate genes that are likely to be causative for PEx. To identify PEx-related pathogenic variants, variants were first filtered to analyze those involved in the regulation of cartilage development [8,9,10], followed by an exome-wide approach to identify damaging variants in genes that are potentially causative for the phenotype.

Following this first analysis of PEx-affected cases, ES was also performed on unaffected family members for variant segregation when available. Genetic variants identified in the first analysis were included for the segregation investigation. Additionally, high-quality variant calls, identified in a curated list of genes associated with cartilage development and/or thoracic aortic aneurysm and dissection using a minor allele frequency (MAF) cutoff of ≤10% and ≤10 homozygotes in gnomAD, were used to identify candidate variants associated with PEx (Supplementary Table S1) [9,10]. In addition, using the same variant call quality specifications as indicated above, candidate variants were identified across the exome in a multi-tiered approach. Briefly, missense variants in genes intolerant to missense variation (Z > 3), loss-of-function (LoF) variants in genes intolerant to LoF (pLI > 0.9), and splice variants predicted to impact canonical splicing (spliceAI > 0.5) were used. Lastly, the eMedGene AI ‘most likely’ variant list was reviewed. All identified candidate variants were curated and interpreted for causality for PEx by considering their predicted impact on gene function, gene function as it relates to PEx, co-segregation, whenever available, and, lastly, classified following the ACMG/AMP guidelines [11]. All variants were annotated against the MANE Select transcript [12].

## 3. Results

**Figure 1:**
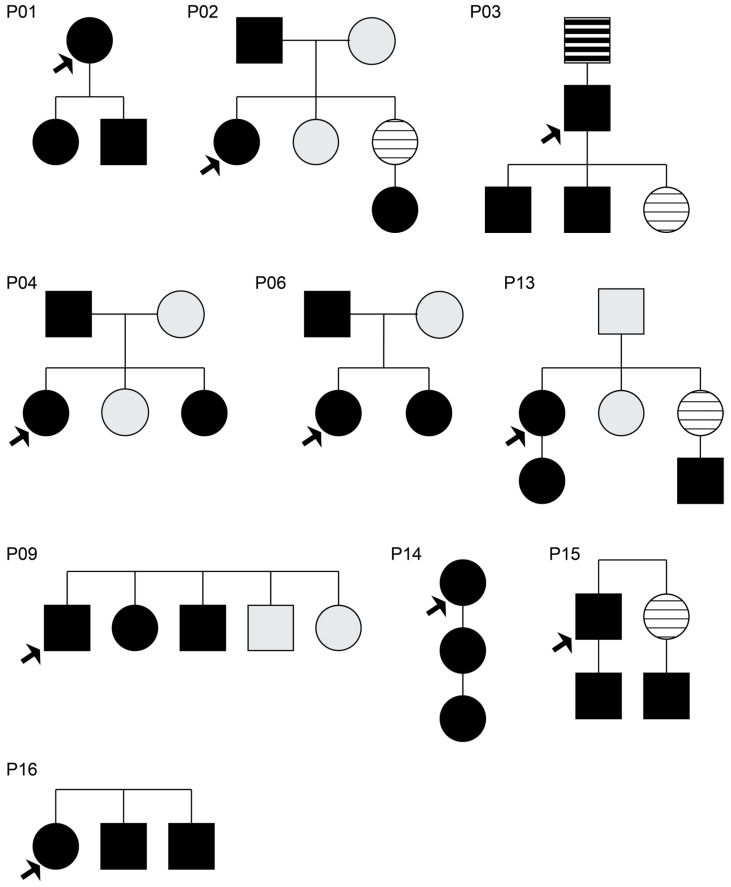
Characteristics of the ten families included in this study. Solid symbols, affected with sequencing data; gray symbols, unaffected with sequencing data; thick dashed symbols, affected without sequencing data; thin dashed symbols, unaffected without sequencing data. Arrows indicate the probands.

In total, ten families with three affected members were included (P01, P02, P03, P04, P06, P09, P13, P14, P15, and P16), resulting in thirty familial PEx cases (Figure 1). Clinical characteristics of the included cohort of PEx patients can be found in Table 1. Different inheritance patterns were represented across the 10 pedigrees, including AD (P01, P02, P03, P04, P06, P13, P14 and P15) and AD or AR (P09 and P16), with possible incomplete penetrance (P02, P13, and P15). In five of these families (P02, P04, P06, P09, and P13), ES was also performed on non-affected members to complete the variant segregation analysis. Families P01, P03, P13, P15, and P16 did not have genetic information available from unaffected members for ES.

### 3.1. Monogenic Analysis of Affected Familial Cases

To identify potential monogenic drivers of familial PEx, our initial analysis focused on genes related to cartilage development (Supplementary Table S1). Among the 30 PEx familial cases, we identified six candidate variants from four families as indicative of a potential monogenic etiology (Table 2).

Among the identified genes, *BMP6* is an inducer of cartilage and bone formation [13]. The BMP6 variant found in P01 (*BMP6* c.379G>A p.(Gly127Arg)) is rare, with moderate to low in silico prediction of a damaging effect. The BMP6 variant observed in P14 (*BMP6* c.720C>G p.Phe240Leu) is also rare, but in silico supports a potential damaging effect.

*NOTCH3* acts as a transcriptional activator of target genes, and its primary expression is in arterial tissue. The variant in P01 (*NOTCH3 c.329G>A p.Arg110His*) was moderately elevated in silico, thus supporting a damaging effect. *CRTAC1* is involved in chondrogenic differentiation upon *BMP4* stimulation, and it is associated with cartilage function. However, the variant observed in P02 (*CRTAC1 c.811C>T p.Arg271Trp*) was conflicting in silico, thus the damaging effect is indeterminate.

*BAZ1B* acts as a transcriptional regulator through chromatin remodeling and has a broad expression profile. In silico analysis of this variant in P03 (BAZ1B c.2134G>A p.Asp712Asn) supports tolerated substitution. Lastly, *GLG1* blinds fibroblast growth factor and E-selectin and has a broad expression profile. However, while low combined annotation-dependent depletion in the variant found in P03 (GLG1 c.2935-54T>C p.?) indicates no deleterious effect, SpliceAI predicts the gain of an in-frame cryptic acceptor splice site, without disruption of the canonical splice sites, to result in the inclusion of 51bps of intron 21. The inclusion of this sequence codes for a premature stop codon (KSFVF—) and is expected to lead to nonsense-mediated decay of the transcript and loss of function. Interestingly, this gene is intolerant to loss of function (pLI = 1). However, this variant may result in incomplete splicing, given its in silico prediction and its minor allele frequency of 0.02% in the gnomAD population.

### 3.2. Segregation Analysis of Affected and Unaffected Family Members

Segregation analysis was performed in five families (P02, P04, P06, P09, and P13), where genetic information was also available for non-affected members. For this investigation, variants previously identified in the monogenic analysis were included. In addition, by leveraging the co-segregation of variants in affected and currently unaffected family members, we revisited any variants that may have been considered non-contributory to their predicted non-deleterious impact.

Considering that genetic information from non-affected members was not obtained from families P01, P03, P14, P15, and P16, the previously identified *BMP6 c.379G>A* and *NOTCH3 c.329G>A* in P01, *BAZ1B c.2134G>A* and *GLG1 c.2935-54T>C* in P03, and *BMP6 c.720C>G* in P15 were not able to be evaluated further for segregation. Thus, only the variant *CRTAC1* c.811C>T p.Arg271Trp identified in family P02 was included in the segregation analysis.

#### 3.2.1. Family P02

This three-generation family was composed of the proband (42-year-old female with severe symptomatic PEx), an affected father, an unaffected mother, an unaffected sister, and an affected niece (Figure 1).

During monogenic analysis, three affected family members were observed to carry *CRTAC1 c.811C>T p.Arg271Trp*. This variant was absent in the unaffected mother, but present in the unaffected sister, so the variant did not segregate with the phenotype. The analyses of three relevant variants that were additionally identified for the segregation analysis are shown in Table 3.

#### 3.2.2. Family P04

This was a two-generation family composed of the proband (21-year-old female with severe PEx), an affected father, an unaffected mother, an unaffected sister, and an affected sister (Figure 1). No variants were identified in the cartilage-development-associated gene slice. Only one variant from the TAAD-associated gene slice segregated with the phenotype (Table 3); however, this pathogenic variant (*COL5A1*, *NM_000093.5:c.-180C>An*) exhibited a low in silico score, therefore suggesting a non-deleterious effect.

#### 3.2.3. Family P06, Family P09, and Family P13

Family P06 was a two-generation family composed of the proband (51-year-old female with severe PEx), an affected father, an unaffected mother, and an affected sister. Family P09 was a one-generation family composed of the proband (55-year-old male with severe PEx), an affected bother, an affected sister, an unaffected bother, and an unaffected sister. Lastly, Family P13 was a three-generation family composed of the proband (55-year-old female with severe PEx), an affected daughter, an affected nephew, an unaffected father, and an unaffected sister (Figure 1). No variants in known genes were found to be strongly associated with PEx within these three families.

## 4. Discussion

Accumulating evidence indicates a likely genetic cause of PEx. Approximately 40% of PEx cases are seen in families and there is a documented association with connective tissue disorders and other genetic syndromes. Despite this, no definite genetic contributor to PEx development has been identified to date and the exact pattern of inheritance has remained elusive [5]. Prior studies in the field of genetic inheritance of PEx have included mainly comprehensive genetic analysis of isolated familial cases, while larger studies only relied on low-resolution approaches [3,5,6]. This study, the largest to date including an ES analysis of multiple PEx families, was performed in an effort to identify potential pathogenic variants associated with the development of PEx.

While our data indicate the absence of a single genetic alteration within the coding part of the genome as a universal driver of familial PEx, they highlight several intriguing family-specific candidates. The *REST* variant found in the P02 family was identified in affected individuals (proband, affected father and niece) and absent in unaffected members (unaffected mother and sister), thus segregating with the condition. *REST* encodes a transcription factor that represses the transcription of neuronal genes in non-neuronal cells, and the lack of functional *REST* results in embryonic lethality during embryonic development [15]. This gene is essential for neuronal development, and a premature loss of *REST* results in the progenitor cells prematurely exiting the cell cycle. The mutation found in the gene *REST* in our cohort has been previously associated with other diseases, such as deafness and gingival fibromatosis [16]. Although none of the PEx cases affected by this mutation in our cohort suffered from any of these concomitant conditions, the presence of pectus deformities has been reported in sporadic familial cases of gingival fibromatosis associated with *REST* variants [14].

The *SMAD4* variant in the P02 family was also identified in affected individuals, and absent in unaffected members. The *SMAD4* missense variant has been described to predispose individuals to thoracic aortic diseases [17]. *SMAD4* pathogenic variants are also associated with syndromic conditions (Juvenile polyposis syndrome and Myhre syndrome). Although features associated with the aforementioned conditions were not seen in the family included in this study, the genetic variant is novel and, as such, it may have a different phenotypic impact. The *CRTAC1* c.811C>T variant, which was observed in each of the three affected family members in the P02 family in the initial analysis, was absent in the unaffected mother but present in the unaffected sister. Nevertheless, under a monogenic disorder, the pedigree indicates incomplete penetrance, given the niece’s mother is also unaffected, which may be indicative that this variant could possibly be relevant. In the P04 family, a 5′UTR variant impacting *COL5A,* associated with Ehlers–Danlos syndrome and Fibromuscular dysplasia, segregated with phenotype. However, this is classified as a likely benign mutation; thus, its clinical relevance seems not apposite.

There are limited data in the literature regarding the genetics and inheritance of PEx. Creswick et al. analyzed the pedigree of 34 families in which PEx was present in two or more members, reporting inconsistent results: while autosomal dominant inheritance was suggested in 14 families, autosomal-recessive inheritance was present in 4, X-linked recessive inheritance was suggested in 6, and 10 families had more complex inheritance patterns. Horth et al. assessed the pedigrees and clinical features of 116 individuals from 56 families [5]. They reported evidence supporting autosomal recessive inheritance and concluded that the regions of Chromosomes 5, 15, and 17 are relevant for candidate gene searches, since genes affecting cartilage are found on these chromosomes.

In a similar fashion to our study, Tong et al. performed whole-exome sequencing in a family with four generations of dominant inherited PEx [6]. In the four affected patients, a novel heterozygous stop-gain variant in Tubulointerstitial Nephritis Antigen (TINAG) was identified, and this variant was not found in the members of the family without the condition. TINAG is a protein-coding gene, and diseases associated with TINAG include membranous nephropathy and interstitial nephritis. Previous studies have demonstrated that TINAG is associated with cell adhesion and the formation of the extra-cellular matrix, too. However, this variant was not confirmed by larger studies, so the direct link between TINAG and PEx could not be confirmed. In another study, Wu et al. sequenced the whole exomes for all four PEx cases and one unaffected individual in a four-generation family affected by PEx [4]. They identified mutations in the sulfotransferase gene GAL3ST4 as the potential cause of PEx and conducted a functional study, which confirmed that this genetic mutation affects the functions of the encoded protein. As can be noticed from the prior literature, there is an evident lack of robust data in regard to ES analysis in PEx families [18,19]. In our study, all GAL3ST4 and TINAG variants identified in the affected probands were considered non-contributory, as they are common in the population or were inherited from an unaffected parent.

Lastly, it is interesting that PEx has been linked to known genetic diseases in prior publications. PEx deformities could indeed be associated with more than 20 genetic conditions, mainly including connective tissue disorders (Marfan syndrome and Ehlers–Danlos syndrome), chromosomal disorders, neurologic disorders, and syndromic disorders [20]. Notably, in Marfan syndrome, PEx is considered a clinical manifestation of the inherited fibrillin-1 deficiency [21]. However, most of the PEx cases (both familial and sporadic cases) are non-syndromic and are most often isolated, further supporting the idea that the exact pattern of inheritance is complex and multifactorial.

Limitations of this study include its small sample size (ten families) and the absence of genetic information from unaffected members in some families, although ours is the largest genetic study of PEx utilizing ES. Expanding this type of analysis to a broader PEx population with a robust representation of both affected and unaffected family members has the potential to increase our understanding of this disease. Another important limitation of our study relates to the sequencing approach used. ES is a very robust method for the identification of genetic alterations in the protein-coding part of the genome. However, the coverage of regulatory aspects within the exome is inadequate for the assessment of potential recurrent alterations in regulatory regions. Furthermore, since the exome represents approximately only 1.5% of the entire genome, our study cannot exclude important variants beyond the exome, which could impact crucial developmental processes and contribute to typical PEx phenotype development. Genetic alterations such as large structural changes and/or rearrangements or deep intronic variants were not part of our analysis and may warrant review in a future study.

In summary, while prior studies have hypothesized either a monogenic or polygenic etiology to PEx, our in-depth analysis across ten families, with multiple affected members per family, to identify a monogenic gene variant did not reveal a strong genetic causality. These findings, while negative, do support a multi-factorial genetic cause for PEx that has yet to be revealed. Additionally, our findings suggest several potentially relevant variants, paving the way for new studies utilizing novel technologies or polygenic evaluations. Our overall findings correlate with prior studies that reported divergent and inconclusive results in the genetic inheritance of PEx, thus supporting the complexity of genetic variants and inheritance modes of pectus deformities [3].

## 5. Conclusions

Among ten families with at least three affected members with pectus excavatum, no definitive shared candidate genes were identified to be both causative for PEx or strongly associated with this disease. However, several variants segregate with the condition in individual families, highlighting the need for future studies in the field. These findings align with the current limited literature that suggests there is no single genetic or chromosomal defect responsible for PEx. Future studies examining the unexplored parts of the genome, including epigenetic components, as well as assessment of multifactorial processes that may contribute to the development of PEx, are warranted.

## Figures and Tables

**Figure 2:**
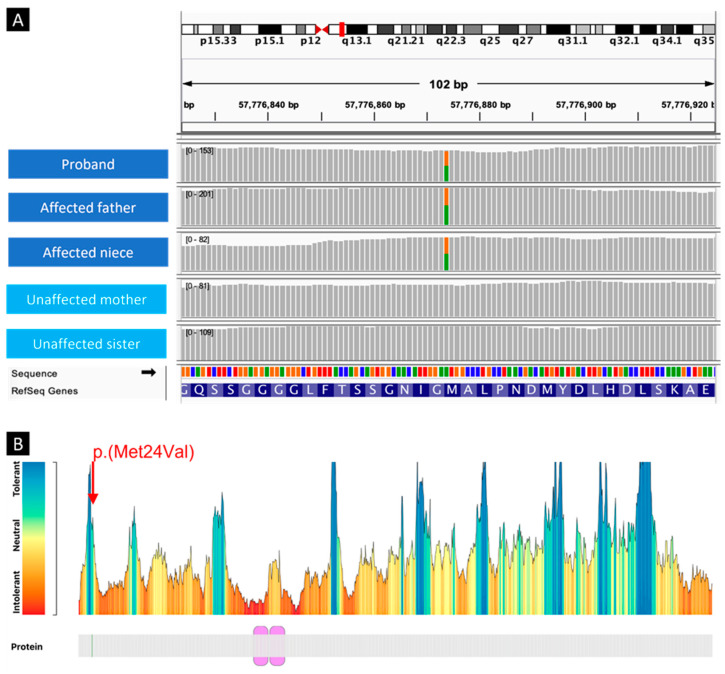
Genetic analysis of the REST mutation (REST, NM_005612.5:c.70A>G, NP_005603.3:p.Met24Val) found in family P02. Variant segregates with phenotype (**A**), but further analyses indicate that substitution is tolerated (**B**), thus suggesting a limited damaging effect of this variant.

**Table 1:** Clinical characteristics of the ten included probands, including disease severity.

Patients	Brief Clinical Presentation and Course
Proband 01	This is a 57-year-old female who was found to have a mildly symptomatic pectus excavatum deformity with right heart compression. Patient’s CT findings revealed a Haller Index of 3.25, which worsens to 3.43 with expiration.
Proband 02	This is a 40-year-old female with a history of severe and symptomatic pectus excavatum (Haller Index 3.8) who underwent minimally invasive repair. The patient underwent bar removal 3 years following initial repair.
Proband 03	This is a 47-year-old male with a history of severe symptomatic pectus with a Haller Index of 3.51 and evidence of right heart compression. The patient underwent a minimally invasive repair of pectus excavatum followed by bar removal after 3 years.
Proband 04	This is a 19-year-old female with a history of severe symptomatic pectus excavatum with a Haller Index of 6.8 who underwent minimally invasive repair of PEx. She underwent bar removal after 3 years with no complications.
Proband 06	This is a 49-year-old female with a history of severe symptomatic pectus with a Haller Index of 5.5 and evidence of right heart compression. The patient underwent minimally invasive repair of pectus excavatum followed by bar removal and bone osteophyte excision after 3 years.
Proband 09	This is a 53-year-old male with a history of severe symptomatic asymmetrical pectus with a Haller Index of 10.0 on inspiration, and a cardiac compression index of 5.79. The patient underwent hybrid minimally invasive repair of pectus excavatum followed by bar removal after 3 years.
Proband 13	This is a 42-year-old female with a history of severe symptomatic pectus with a Haller Index of 5.5 and evidence of right heart compression. The patient underwent minimally invasive repair of pectus excavatum followed by bar and bone osteophyte removal after 3 years.
Proband 14	This is a 58-year-old female with a history of severe symptomatic pectus with a Haller Index of 5.4 and evidence of right heart compression. The patient underwent hybrid minimally invasive repair of pectus excavatum followed by bar and bone osteophyte removal after 3 years.
Proband 15	This is a 49-year-old male with a history of severe worsening symptomatic pectus with a Haller Index of 5.67 on inspiration and 9.2 on expiration and evidence of right heart compression. The patient underwent hybrid minimally invasive repair of pectus excavatum followed by bar and bone osteophyte removal after 3 years with no complications.
Proband 16	This is an 18-year-old female with a history of severe symptomatic pectus excavatum with a Haller Index of 4.6 and evidence of right heart compression. The patient underwent minimally invasive repair of pectus excavatum followed by bar and bone osteophyte removal after 3 years with no complications.

**Table 2:** Monogenic analysis of affected members in PEx families.

Family	Gene/Variant	Zygosity	In Silico	ACMG Classification: Criteria Applied
**PO1**	***BMP6*** c.379G>A p.Gly127Arg	HET	CADD: 23.8 REVEL: 0.161	VUS: PM2_supp, BP4
	***NOTCH3*** c.329G>A p.Arg110His	HET	CADD: 22.4 REVEL: 0.580	VUS: PM2_sup
**PO2**	***CRTAC1*** c.811C>T p.Arg271Trp	HET	CADD: 29.5 REVEL: 0.194	VUS: PM2_supp, BP4
**PO3**	***BAZ1B*** c.2134G>A p.Asp712Asn	HET	CADD: 22.5 REVEL: 0.107	VUS: PM2_supp, BP4_mod
	***GLG1*** c.2935-54T>C p.?	HET	CADD: 5.78 SPLICEAI: AG = 0.33 (AP = −3 bp)	VUS: PM2_supp, PP3
**P14**	***BMP6*** c.720C>G p.Phe240Leu	HET	CADD: 25.5 REVEL: 0.857	VUS: PM2_supp, PP3_mod

**HET**: heterozygous; **CADD**: Combined Annotation Dependent Depletion, v1.6; **REVEL**: rare exome variant ensemble learner; **VUS**: variant of uncertain significance.

**Table 3:** Summary of the genetic variants that segregate with phenotype in the familial analysis.

Gene/Variant	Gene Slice	Family	OMIM Disease	Zygosity	In Silico	Conclusions	ACMG Classification: Criteria Applied
*SMAD4* NM_005359.6:c.-69G>A	**TAAD**	**P02**	**Myhre syndrome** (MIM: 139210) [AD] **Juvenile polyposis/hereditary hemorrhagic telangiectasia syndrome** (MIM: 175050) [AD]	**proband: HET** **father: HET** mother: REF sister: REF **niece: HET**	CADD: 5.948 REVEL: NA SPLICEAI: 0.01	Rare variant that segregates with phenotype. The low CADD suggested a non-deleterious effect.	VUS: PM2_supp, BP7
*COL5A1,NM_000093.5:c.-180C>A*	**TAAD**	**P04**	**Ehlers-Danlos syndrome, classic type, 1** (MIM: 130000) [AD] **Fibromuscular dysplasia, multifocal** (MIM: 619329) [AD]	**proband: HET** **father: HET** mother: REF **affected sister: HET** unaffected sister: REF	CADD: UNK REVEL: NA SPLCEAI: 0	Variant segregates with phenotype. The low CADD suggested non-deleterious effect.	B: BS2, BP7
*ASXL3,* *NM_030632.3:c.6236T>C,* *NP_085135.1:p.Ile2079Thr*	**Exome-wide candidate list.**	**P02**	**Bainbridge-Ropers syndrome** (MIM: 615485) [AD]	**proband: HET** **father: HET** mother: REF sister: REF **niece: HET**	CADD: 25.5 REVEL: 0.29	This variant segregates with phenotype. Differential strongly indicates that this variant is non-contributory to disease development.	VUS: PM2_supp
*REST*, NM_005612.5:c.70A>G, NP_005603.3:p.Met24Val	**Exome-wide candidate list.**	**P02**	**Deafness, autosomal dominant 27** (MIM: 612431) [AD] **Fibromatosis, gingival, 5** (MIM: 617626) [AD]	**proband: HET** **father: HET** mother: REF sister: REF **niece: HET**	CADD: 21.1 REVEL: 0.18	Variant segregates with phenotype (Figure 2A). In silico tools indicate that substitution is tolerated (Figure 2B); however, pathogenic variants in REST are associated with loss-of-function mechanisms. This variant has been associated with pectus deformities in prior studies [14,15]	B: BP4, BS2

**TAAD**: genes associated with thoracic aortic aneurysm and dissection; **OMIM**: Online Mendelian Inheritance in Man; **AD**: autosomal dominant; **HET**: heterozygous; **CADD**: combined annotation-dependent depletion, v1.6; **REVEL**: rare exome variant ensemble learner; **UNK**: unknown; **NA**: not applicable; **VUS**: variant of uncertain significance; **B**: benign.

## Data Availability

The data presented in this study are available on request from the corresponding author.

## Supplementary Materials

The following supporting information can be downloaded at https://www.mdpi.com/article/10.3390/genes15111429/s1: Table S1: Genes analyzed for high quality variants.
