# Supplementary material for: The Complexity of Familial Inheritance in Pectus Excavatum: A Ten-Family Exome Sequencing Analysis
Source: Genes (Basel). 2024 Nov 1;15(11):1429. doi: 10.3390/genes15111429 (PMC11593651; doi:10.3390/genes15111429)
Supplement: Supplementary file 1 [file genes-15-01429-s001.zip › genes-3257353-supplementary.pdf]

Supplementary Table 1. Genes analyzed for high quality variants.

**Variants in genes indicated to be associated to cartilage development**<br>*(7 genes)*	ACAN, COL2A1, FBN2, TGFB, TGFBR1, TGFBR2, TGFBR3
**Variants impacting thoracic aortic aneurysm and dissection (TAAD) associated genes**<br>*(59 genes)*	ABL1, ACTA2, BGN, CBS, COL3A1, COL5A1, COL5A2, EFEMP2, ELN, FBLN5, FBN1, , FKBP14, FLNA, FOXE3, LOX, MFAP5, MYH11, MYLK, NOTCH1, PLOD1, PRKG1, SKI, SLC2A10, SMAD2, SMAD3, SMAD4, SMAD6, TGFB2, TGFB3, ABCC6, ACVR1, ADAMTS2, ALDH18A1, ATP6V0A2, ATP7A, B4GALT7, CHST14, COL11A1, COL11A2, COL1A1, COL1A2, COL2A1, COL4A1, COL9A1, COL9A2, COL9A3, EMILIN1, FLCN, HNRNPK, KCNN1, LTBP2, MAT2A, MED12, MYLK2, PKD1, PKD2, SLC39A13, TNXB, ZNF469
